# Treatment preferences by disease severity in alopecia areata: A cross-sectional survey study

**DOI:** 10.1016/j.jdin.2025.11.023

**Published:** 2025-12-12

**Authors:** Ursula Biba, Katherine Sanchez, Samantha Gregoire, Natasha A. Mesinkovska, Monique Waldman, Lisa Anderson, Arash Mostaghimi

**Affiliations:** a David Geffen School of Medicine at University of California Los Angeles, Los Angeles, California; b Department of Dermatology, Brigham and Women’s Hospital, Boston, Massachusetts; c Lake Erie College of Osteopathic Medicine, Erie, Pennsylvania; d University at Buffalo Jacobs School of Medicine and Biomedical Sciences, Buffalo, New York; e National Alopecia Areata Foundation, San Rafael, California; f Department of Dermatology, University of California Irvine School of Medicine, Irvine, California; g Department of Dermatology, Columbia University, New York, New York

**Keywords:** alopecia areata, attributes, hair loss, preferences, treatments, values

*To the Editor:* Alopecia areata (AA) is an autoimmune condition that manifests in unpredictable areas of nonscarring hair loss.^1^ Effective regimens are especially challenging for individuals with severe AA, who report low treatment satisfaction.^1^ While individuals with AA have deemed treatment efficacy important, the impact of AA severity on treatment preferences has not been well described.^2^^,^^3^ We aim to evaluate preferred treatment attributes among individuals with mild, moderate, and severe AA.

Participants recruited from the National Alopecia Areata Foundation completed a cross-sectional questionnaire between November 2022 and January 2023 (response rate: 58%). US citizens aged 18 years and over with diagnosed AA who had experienced hair loss and seen a provider in the last year were included. The primary outcome of this study was treatment attribute rating, while secondary outcomes were differences in ratings based on overall AA severity. Kruskal-Wallis and post hoc Dunn tests were performed to identify differences between groups. Ordered logistic regressions were performed to identify any effects on attribute rankings stemming from participant demographics (Supplementary Table I, available via Mendeley at https://data.mendeley.com/datasets/fwf25p37r8/1).

Of the 500 respondents, most were female (*n* = 431, 86.2%), White (*n* = 400, 80.0%), insured (*n* = 480, 96.0%), and diagnosed with severe AA (*n* = 331, 66.2%) (Table I). Mean ratings for treatment preferences were highest for hair regrowth (6.51 ± 1.28), insurance coverage (6.18 ± 1.56), and preventing AA flares (6.10 ± 1.28) (Table II). Treatment frequency ratings differed between those with mild and severe AA (4.95 vs 3.72; *P* = .0079) and moderate and severe AA (4.16 vs 3.72; *P* = .0253), with individuals with severe AA rating frequency as less important (Table II). Logistic regression controlling for age, sex, education level, annual income, and health insurance status did not identify significant effects on treatment attribute ratings, except for being health insured leading to higher value in insurance mitigating AA treatment costs (odds ratio: 5.34; *P* = .020; Supplementary Table I, available via Mendeley at https://data.mendeley.com/datasets/fwf25p37r8/1).

**Table I:** Participant demographics, AA clinical characteristics, and self-reported current AA treatments

Participant demographics and AA characteristics	All participants (*N* = 500)	Mild AA (*n* = 21)	Moderate AA (*n* = 148)	Severe AA (*n* = 331)
Age, mean (SD)	50 (14.6)	47.5 (14.9)	49.4 (15.2)	50.4 (14.3)
Female sex assigned at birth, *n* (%)	431 (86.2)	19 (90.5)	132 (89.2)	280 (84.6)
Hispanic ethnicity, *n* (%)	54 (10.9)	3 (14.3)	25 (16.9)	26 (7.9)
Non-Hispanic ethnicity, *n* (%)	441 (89.1)	18 (85.7)	122 (82.4)	301 (90.9)
Racial identity, *n* (%)				
White	400 (80.0)	14 (66.7)	120 (81.1)	266 (80.4)
Black	47 (9.4)	3 (14.3)	12 (8.1)	32 (9.7)
Annual income, *n* (%)				
<$25,000	44 (8.8)	2 (9.5)	11 (7.4)	31 (9.4)
$25,000-$49,999	80 (16.0)	4 (19.1)	25 (16.9)	51 (15.4)
$50,000-$74,999	73 (14.6)	6 (28.6)	16 (10.8)	51 (15.4)
$75,000-$99,999	67 (13.4)	3 (14.3)	22 (14.9)	42 (12.7)
≥$100,000	171 (34.2)	1 (4.8)	55 (37.2)	115 (34.7)
Education level, *n* (%)				
High school graduate or equivalent∗	155 (31.0)	8 (38.1)	43 (29.1)	104 (31.4)
College graduate	173 (34.6)	10 (47.6)	60 (40.5)	103 (31.1)
Completed graduate school	157 (31.3)	2 (9.5)	43 (29.1)	112 (33.8)
Health-insured, *n* (%)	480 (96.0)	21 (100.0)	143 (96.6)	316 (95.5)
Formal diagnosis of anxiety, *n* (%)	216 (43.2)	9 (42.9)	57 (38.5)	150 (45.3)
Formal diagnosis of depression, *n* (%)	156 (31.2)	7 (33.3)	39 (26.4)	110 (33.2)
Aware of new AA treatments, *n* (%)	280 (56.0)	11 (52.4)	80 (54.1)	189 (57.1)
Self-reported current AA treatments, *n* (%); *n* = 909†				
Steroid/corticosteroid injections	118 (13.0)	8 (38.1)	48 (32.4)	62 (18.7)
Topical steroids/corticosteroids‡	128 (14.1)	7 (33.3)	52 (35.1)	69 (20.8)
Oral steroids	22 (2.4)	1 (4.8)	8 (5.4)	13 (3.9)
Topical minoxidil	86 (9.5)	4 (19.1)	36 (24.3)	46 (13.9)
Oral minoxidil	43 (4.7)	4 (19.1)	9 (6.1)	30 (9.1)
Anthralin cream or ointment	5 (0.6)	-	1 (0.7)	4 (1.2)
Topical immunotherapy§	13 (1.4)	1 (4.8)	2 (1.4)	10 (3.0)
Oral immunomodulators‖	113 (12.4)	1 (4.8)	31 (21.0)	81 (24.5)
Over-the-counter agents¶	193 (21.2)	14 (66.7)	73 (49.3)	106 (32.0)
Other	67 (7.4)	5 (23.8)	27 (18.2)	35 (10.6)
None	121 (13.3)	1 (4.8)	18 (12.2)	102 (30.8)

*AA*, Alopecia areata.

∗ Equivalent degree defined as: associate’s degree, vocational school, or certificate.

† Total treatments reported do not add to 500 because participants were given the option to select multiple treatments. Percentages are reported using 909, the total responses, as the denominator.

‡ Administered as lotions, foams, creams, or ointments.

§ Defined as diphencyprone [DPCP], dinitrochlorobenzene [DNCB], or squaric acid dibutyl ester [SADBE].

‖ Defined as tofacitinib [Xeljanz], ruxolitinib [Jakafi], or baricitinib [Olumiant].

¶ Defined as nonprescription medication(s), nutritional supplements, or vitamins.

**Table II:** Treatment attributes by mean (SD) ranking in the whole sample and stratified by AA disease severity

Treatment attribute	Mean (SD)			
	Whole sample	Mild AA	Moderate AA	Severe AA
Hair regrowth efficacy	6.51 (1.28)	6.81 (0.51)	6.48 (1.32)	6.50 (1.29)
Insurance coverage mitigating treatment costs	6.18 (1.56)	6.38 (1.36)	6.05 (1.66)	6.23 (1.53)
Efficacy at preventing AA flare-ups	6.10 (1.58)	6.57 (0.82)	6.00 (1.64)	6.12 (1.59)
Side effects	5.92 (1.62)	6.10 (1.48)	6.04 (1.46)	5.86 (1.70)
Price	5.87 (1.62)	6.00 (1.30)	5.76 (1.66)	5.92 (1.62)
Contraindications	5.28 (1.92)	5.67 (1.71)	5.29 (1.91)	5.25 (1.94)
Treatment location	4.42 (2.16)	4.76 (2.12)	4.54 (2.08)	4.35 (2.21)
Treatment length	4.40 (2.18)	4.81 (1.91)	4.66 (2.09)	4.26 (2.23)
Oral medication	4.03 (2.41)	4.62 (2.20)	4.05 (2.41)	3.98 (2.42)
Injectable medication	3.94 (2.30)	3.67 (2.29)	4.13 (2.35)	3.88 (2.28)
Topical medication	3.94 (2.29)	4.19 (2.16)	3.92 (2.30)	3.93 (2.30)
Treatment frequency	3.90∗ (2.27)	4.95 (2.04)	4.16 (2.22)	3.72†^,^‡ (2.29)

Participants rated treatment attributes on a scale of 1 to 7, with 1 being least impactful and 7 being most impactful to the likelihood of using a potential treatment. Treatment frequency was the only attribute that differed by disease severity.

*AA*, Alopecia areata.

∗ Significant Kruskal-Wallis result (*P* = .014).

† Significant post hoc Dunn test result versus mild AA (*P* = .008).

‡ Significant post hoc Dunn test result versus moderate AA (*P* = .025).

When selecting potential treatments, patients with AA valued hair regrowth efficacy, flare prevention, and coverage by insurance most. These preferences were maintained across disease severity and baseline demographics, consistent with prior studies.^2^^,^^4^^,^^5^ If treatments are affordable and effective, individuals with AA are flexible with treatment length, frequency, and route of administration.

This study is limited by low sample diversity, limiting generalization to men or individuals outside the United States, and high baseline AA-related health literacy, given that inclusion criteria selected for participants with an official AA diagnosis who had recently seen a provider. Revisiting these findings once additional therapeutic options are available may reveal an evolution in patient preferences. Furthermore, this study did not ask participants how the availability, or lack thereof, of treatment options affects the experience of managing AA.

Future study is needed to connect the experience of navigating the health system for effective AA therapies to quality of life and psychological impact measures. Larger sample sizes of patients with mild, moderate, and severe AA are needed to further uncover and understand trends between disease states. Until then, continued focus on overall efficacy and financial accessibility is key for promoting patient-centered treatments.

## Conflicts of interest

Dr Mostaghimi has received consulting fees from AbbVie, ACOM Health, Bioniz, Boehringer Ingelheim, Concert, Digital Diagnostics, Eli Lilly, Equillium, Hims & Hers Health, and Pfizer; equity from ACOM Health, Figure 1, and Hims & Hers Health; licensing/royalties from Concert and Pfizer; and research funding from Aclaris, Concert, Eli Lilly, and Incyte. Other authors have no conflicts of interest to disclose.
